# Cultural and Traditional Dietary Practices Among African Pregnant Women: A Scoping Review

**DOI:** 10.7759/cureus.97508

**Published:** 2025-11-22

**Authors:** Pinelopi Varela, Maria Bouroutzoglou, Christina I Nanou, Paraskevi Papadogeorgou, Elena Klara Stamouli, Anna Deltsidou

**Affiliations:** 1 Department of Neonatology, General Hospital of Athens “Alexandra”, Athens, GRC; 2 Department of Midwifery, International Hellenic University, Thessaloniki, GRC; 3 Department of Midwifery, University of West Attica, Athens, GRC

**Keywords:** african pregnant women, cultural dietary practices, food taboos, midwives, nutrition during pregnancy

## Abstract

Pregnant women’s dietary practices play an important role in maternal and child well-being. WHO’s statement emphasizes the importance of a varied diet for pregnant women. The literature indicates that, due to traditional cultural beliefs, pregnant women in Africa frequently refrain from consuming certain nutrient-dense foods. This scoping review aimed to map the existing literature on cultural and/or traditional dietary practices among African pregnant women and to identify the underlying motivations that shape these practices. A systematic search was performed using the Population, Concept, and Context (PCC) framework in three electronic databases. Articles were screened for eligibility and summarized through a narrative synthesis. The review structure complies with the Preferred Reporting Items for Systematic Reviews and Meta-Analyses extension for Scoping Reviews (PRISMA-ScR) guidelines. Thirteen studies published within the period from 2012 to 2024 met the inclusion criteria and were included in this review. These studies were out of five African nations, such as Nigeria, Ethiopia, Malawi, the Democratic Republic of the Congo, and Uganda. Sample sizes ranged from nine to 422 participants. The majority of studies (n = 11) reported that participants avoided protein-rich foods, followed by avoidance of dairy products, vegetables, and fruits. The most common reason for maintaining food taboos among African pregnant women was the belief that consuming these nutritious foods could lead to complications during labor or negatively affect the newborn. Food taboos among African pregnant women exist and include foods that are high in protein, fruits, and vegetables. In midwifery clinical settings, healthcare professionals need to approach these dietary practices with cultural sensitivity to empower African pregnant women to achieve a balance between tradition and the needs for maternal and child well-being.

## Introduction and background

The maternal nutrition during pregnancy impacts the woman and the fetus in several ways. It lays the groundwork for the mother’s and child’s long-term health while having a major impact on the growth and development of the fetus [1,2]. As a result of pregnancy, the physical demands increase, which in turn requires a decent energy level in the mother and a balanced energy expenditure, which means an adequate caloric intake coming from a balanced and sufficient nutrition [2-4]. It is vital in pregnancy to ensure that the mother gets proper energy in all the stages of pregnancy to ensure the fetus gets all the needed energy and nutrients to grow and develop [5]. According to WHO, pregnant women should adhere to a diversified dietary pattern incorporating animal- and plant-based protein sources such as meat, fish, legumes, and nuts, as well as whole grains, fruits, and green and orange vegetables. Furthermore, adequate intake of critical micronutrients, including folic acid, iodine, calcium, vitamin D, vitamin B12, vitamin B6, vitamins A, E, and K, choline, copper, magnesium, sodium, and zinc, is strongly recommended to support favorable pregnancy outcomes and reduce the risk of maternal and neonatal complications [6-8]. However, some pregnant women often fail to maintain healthy dietary patterns that meet their growing needs because they avoid certain foods [9,10].

Cultural customs are one of the many elements that influence an individual’s eating habits. Environmental, economic, and sociocultural factors all have an impact on dietary behaviors, which include the processes of choosing foods, patterns of consumption, and total nutrient intake. These actions are the result of a complicated interaction between several food-related behaviors that are controlled by a number of interconnected elements [11,12].

One of the dietary behaviors observed is food taboos. Food taboos arise from historical and cultural traditions resulting from social and religious practices whereby eating and drinking certain foods is forbidden. These social customs are unspoken regulations of eating behaviors within a community where particular foods and/or food combinations are deemed unsuitable and unfit for consumption. Such taboos are documented across nearly all human societies and constitute a systematized framework of beliefs and practices that govern food avoidance, frequently linked to cultural identity, hygiene, or spiritual considerations [13,14]. In most cultures, restricting food during pregnancy is a well-known practice, and maternal diet is shaped by various food taboos. These taboos, which are a part of cultural heritage and are often handed down from generation to generation, are concerned with expectant mothers, which in turn determines the type of diet followed during pregnancy [9,15,16]. Pregnancy-related food taboos of religious and cultural origin are often based on actions and concepts that are thought to safeguard both the mother and the fetus.

Abstaining from certain foods is perceived as a protective measure for the mother and has negative consequences for the fetus [13,15-17]. Exclusionary dietary behaviors adopted during pregnancy may also result in limited intake of vital micronutrients. This may cause various kinds of maternal and fetal malnutrition, including deficits in protein, vitamins, and iron [18-20]. A growing body of evidence has linked insufficient maternal nutrition during pregnancy identified as a possible risk factor for poor pregnancy management and negative pregnancy outcomes. Obstructed and prolonged labor, elevated maternal mortality, low birth weight, intrauterine growth restriction (IUGR), preterm birth, and neural tube defects are some of the pregnancy complications and birth defects that are a result of inadequate maternal nutrition [2,21,22].

According to the literature, Africa is among the regions where pregnant women commonly avoid the consumption of certain nutritious foods as a result of traditional cultural beliefs [9,23-26]. It is also among the continents with significant immigration rates within the broader context of global migration flows. The number of foreign migrants worldwide has grown at a rate that is almost twice as fast as the rate of population growth since the turn of the 21st century. Approximately 55 million people, or 20% of all international migrants worldwide, currently reside in European Union member states. The number of refugees worldwide has increased by more than 10 million in the last three years [27]. In 2020, Africa accounted for 40.6 million international migrants, representing 14.5% of the global migrant population, with 27.2% of these migrants residing in Europe. Gender distribution is relatively balanced, with women constituting almost half of African migrants; in 2024, females represented 47.1% of international migrants originating from Africa [28,29].

Given the aforementioned factors, midwifery-related healthcare professionals may be expected to oversee and manage dietary patterns among African pregnant women that involve the avoidance of particular foods or beverages. Understanding the food taboos observed by African pregnant women is therefore crucial for midwifery experts, as it can facilitate the delivery of focused prenatal nutritional counseling. Combining the knowledge of midwifery specialists on these dietary taboos with the provision of tailored prenatal nutritional guidance can enhance this ethnic group’s understanding of nutrition throughout pregnancy. Promoting healthy dietary behaviors, improving the general quality of pregnant women’s diets, and ensuring successful pregnancies all depend on having a solid understanding of nutrition [30,31].

The aim of the present scoping review is to systematically map the existing literature on cultural and/or traditional dietary practices among African pregnant women and to identify the underlying motivations that shape these practices.

## Review

Methods

This scoping review was conducted in accordance with the Preferred Reporting Items for Systematic Reviews and Meta-Analyses extension for Scoping Reviews (PRISMA-ScR) guidelines [32]. Although the research protocol was developed in advance, it was not registered; therefore, a registration number is not available.

Eligibility Criteria

Studies examining traditional or cultural dietary practices among pregnant African women living in African nations were the main focus of this review. Based on the Population, Concept, and Context (PCC) framework, the search strategy applied the following conceptual filters: Context: community-based, clinical, rural, or urban settings throughout Africa; Concept: traditional or cultural dietary beliefs, practices, and taboos; and Population: pregnant women in African countries. Studies were included if they were published in peer-reviewed English-language journals between 2000 and 2024. Studies were included if they focused on pregnant populations in Africa and reported, described, or analyzed cultural or traditional dietary practices during pregnancy. Without regard to geographic location within the African continent, all study designs, such as qualitative, quantitative, or mixed methods, were taken into consideration. Studies were excluded if they focused on nonpregnant populations within Africa, addressed only micronutrient supplementation, or examined nutritional aspects without reference to cultural context. The exclusion criteria also included literature reviews, systematic reviews, meta-analyses, and conference proceedings.

Information Sources

A comprehensive search was carried out from 2000 to 2024 across the databases of African Journals Online, PubMed, and Scopus in order to find possibly relevant research studies. From January to March 2025, a thorough database search was carried out, with no eligible studies from 2025 found during the final search.

Search Strategy

The PubMed search strategy utilized the following combination of keywords: ("traditional dietary practices" OR "food taboos" OR "pregnancy diet" OR "maternal food beliefs" OR "cultural food practices") AND ("Africa" OR the names of specific African countries) AND ("pregnant women" OR "maternal health"). A publication date filter was applied to limit the search to studies published between 2000 and 2024. The literature search was conducted by one author (VP) and independently peer-reviewed by another author (BM).

Selection of Sources of Evidence

Following the removal of duplicates, titles and abstracts were independently screened by two reviewers (VP and PP) based on the predefined inclusion criteria. Two additional reviewers (BM and SEK) also conducted independent title and abstract screening to ensure relevance to the topic. Full-text articles of selected citations were then assessed in detail by the same initial reviewers (VP and PP) to determine eligibility. Rationales for excluding studies that did not meet the criteria were documented. At any point during the selection process, differences were settled by discussion; if agreement could not be reached, two additional reviewers (NC and DA) were consulted before a decision was made. Final inclusion in the scoping review was based on mutual agreement among the reviewers regarding each article’s relevance to the topic.

Data Charting Process, Data Items, and Synthesis of Results

Data were extracted from the studies included in the scoping review by two independent reviewers (VP and NC) using a data extraction tool developed specifically for this study. The tool captured detailed information on the participants, concept, and context, including the first author’s name, year of publication, study design, participant details (such as sample size, participant characteristics, and methods used to report dietary practices), geographic context (African country or region and whether the setting was rural or urban), the concept (dietary practices described and cultural motivations), and the study’s conclusion. Any disagreements between the reviewers were resolved through discussion or, when necessary, with input from a third reviewer (DA). All variables and study-related information were compiled into a tabular format. All included studies are summarized through a narrative synthesis, and the mapping of this scoping review is presented in a table.

Results

Selection of Sources of Evidence

**Figure 1:**
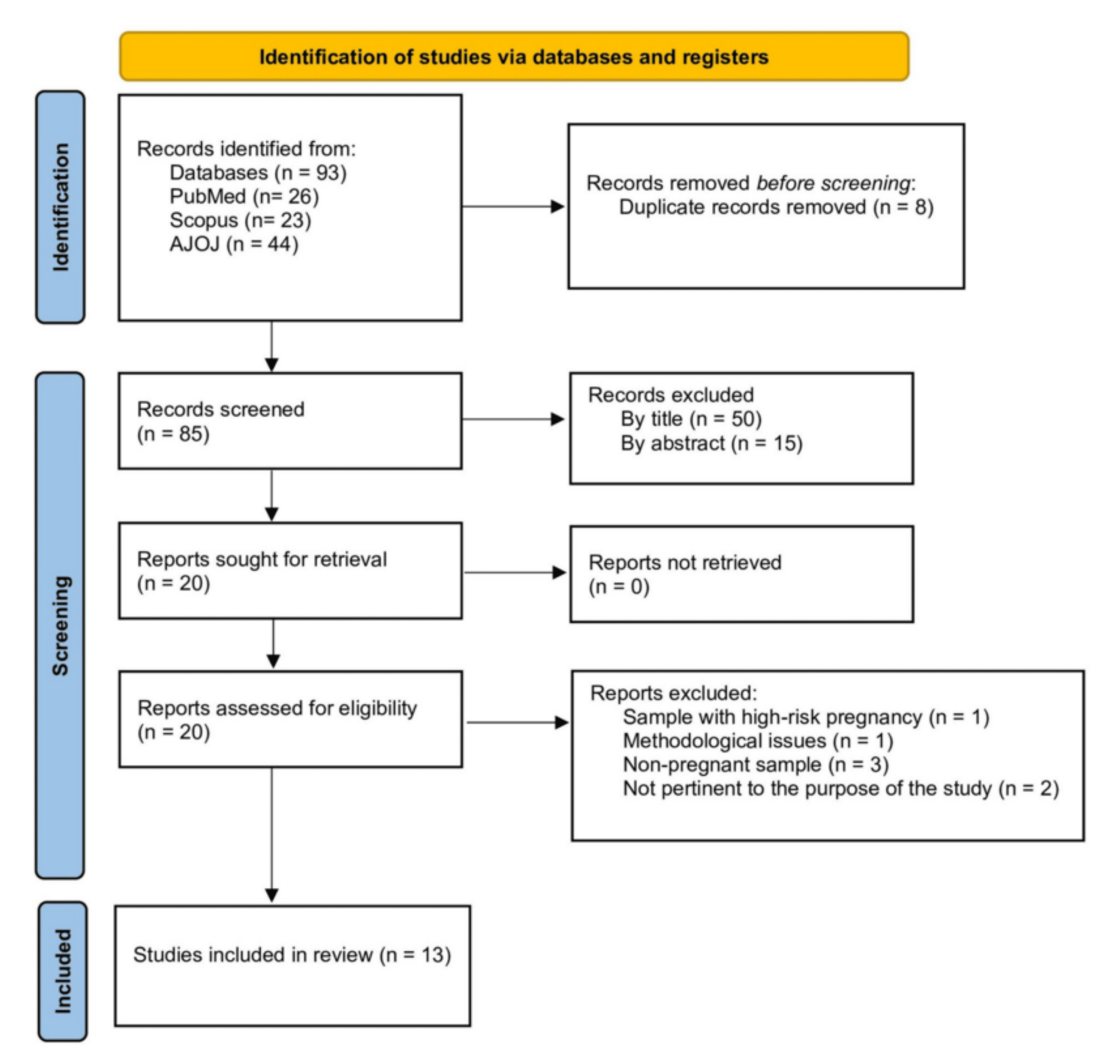
PRISMA flow diagram of included studies PRISMA, Preferred Reporting Items for Systematic Reviews and Meta-Analyses

The process of article identification and selection is illustrated in a PRISMA-ScR flow diagram (Figure 1) [32,33]. After removing duplicates, a total of 85 citations were identified through electronic database searches. Based on title and abstract screening, 65 records were excluded, leaving 20 full-text articles for eligibility assessment. Of these, seven were excluded for the following reasons: one study addressed pregnancy-related pathology and specific dietary practices; one study was excluded due to methodological issues; two studies did not reference traditional or cultural dietary beliefs, practices, or taboos; and three studies involved nonpregnant populations. The remaining 13 studies met the inclusion criteria and were included in this review.

Characteristics of Sources of Evidence

All included studies were published within the period from 2012 to 2024. Among the 13 studies included in the review, five employed a qualitative study design [34-38]. Six studies used a quantitative, cross-sectional design [16,39-43]. The remaining two studies [44,45] applied a mixed-methods approach. All quantitative studies used questionnaires for data collection. Focus group discussions were employed as the data collection method in three qualitative studies [34-36] and in two mixed-methods studies [44,45]. In-depth interviews (IDIs) were used in two qualitative studies [37,38]. Five of the included studies were conducted in Nigeria [16,39,41,42,44], and another five were from Ethiopia [34-36,38,43]. One study was conducted in Malawi [40], one in the Democratic Republic of Congo [37], and one in Uganda [45]. Eight studies were conducted in rural settings [34-36,38-40,44,45]. Three studies took place in urban settings [16,41,42], while two studies were conducted in mixed settings [37,43]. Sample sizes ranged from nine participants [37] to 422 participants [43]. Two studies did not report the number of pregnant participants [34,35]. Most participants were between 19 and 42 years of age, with the exception of one study that reported a lower mean age of 17.7 years [40]. The majority of participants were married across nearly all studies. Educational levels varied significantly: many participants had low or no formal education [34-40,42,44,45], while a few studies reported higher education levels, including college or university attainment [16,41,43]. Most women had experienced between two and five previous pregnancies [16,35,39,42,44], with one study reporting particularly high parity [43] (Table 1).

**Table 1:** Data extraction of included studies FGD, focus group discussion; IDIs, in-depth interviews

Author and year	Study	Country/region (setting)	Sample size	Participants' characteristics	Method of reporting	Described dietary practices	Cultural motivations	Conclusion
Ekwochi et al. (2016) [16]	Quantitative	Nigeria – Enugu (urban)	149	The majority of participants (39.6%) were between 26 and 30 years old, 64.8% had experienced two to five pregnancies, and 51% had attained university-level education.	Questionnaire	A total of 36.5% admitted to avoiding certain foods in pregnancy based on beliefs about food taboos. Commonly, dietary avoidance of snails and grass-cutter meat is practiced.	Snails → drooling; grass-cutter meat → prolonged labor	Food taboos persist in pregnancy. Myths should be addressed during antenatal care.
Zerfu et al. (2016) [34]	Qualitative	Ethiopia – Oromia Region (rural)	Not specified	Mostly from farming households, with low income and high illiteracy	FGDs	The consumption of meat, fish, fruits, and some vegetables during pregnancy remained as low as the pre-pregnancy state, irrespective of the women’s income and educational status. Low intake of fruits, vegetables, meat, and dairy. Dietary avoidance of green leafy vegetables, yogurt, cheese, sugarcane, and green pepper.	Beliefs about some foods (e.g., leafy greens, dairy) sticking to the fetus’s head; sugarcane and fruits are linked to labor risks	Misconceptions about food taboos were widespread. Calls for dietary diversification and education.
Hadush et al. (2017) [35]	Qualitative	Ethiopia – Afar Region (mostly rural)	Not specified	Participants were aged between 19 and 39 years, had a low level of education, and had more than two children.	FGDs	Dietary avoidance of solid, fatty (meat, milk, and yogurt), and cold foods	Prevent difficult labor, fetal overgrowth, bleeding during labor, disease, and discoloration of the skin of the child	Taboos widely restrict food during pregnancy.
Tsegaye et al. (2021) [36]	Qualitative	Ethiopia – Illu Aba Bor Zone (rural)	26	Mostly housewives with no formal education (51.9%).	FGDs	Dietary avoidance of vegetables (cabbage and pumpkin), milk and milk products, eggs, sugarcane, and fruits (bananas and avocado)	Beliefs about plastered fetal heads, large neonates, and childbirth complications	Need to strengthen antenatal nutrition education.
Maykondo et al. (2022) [37]	Qualitative	Democratic Republic of Congo – Kwango Province (rural and peri-urban)	9	The participants had a mean age of 23.3 years; most were housewives, had six to 12 years of education, and were in the second or third trimester of pregnancy.	IDIs	Regular consumption of cassava flour, cassava leaves, cowpeas, and amaranths; low fruit and protein intake. Food taboos included pork, eggs, chicken, cowpeas, certain fish species, goat meat, mushrooms, and many others.	Beliefs link food taboos to avoiding large neonates or childbirth complications. Pork meat → pork-like cries in neonates; eggs → absence of hair in children; sorrel leaves → deficiency of breast milk; mushrooms → risk of malnutrition in children; duck meat → yellow fever; chicken → epilepsy in children; red fruits and vegetables → the neonate will have a red anus.	Poor dietary practices risk malnutrition. Multi-pronged education is needed.
Eyasu et al. (2022) [38]	Qualitative	Ethiopia – Tigray Region (rural)	10	The participants were aged 21-42 years old, mostly illiterate (62%), and married (90%).	IDIs	Dietary avoidance of “kollo” (roasted wheat or chickpea), eggs, mustard, and green pepper	Beliefs link food taboos to avoiding cramps, large neonates, dehydration, and delayed childbirth.	Cultural beliefs contribute to undernutrition in pregnancy; calls for culturally sensitive education and awareness.
Ugwa (2016) [39]	Quantitative	Nigeria – Kano State (rural)	200	The participants had a mean age of 23.7 years; most were uneducated and married, 51% were unemployed, and 54.5% had one to four children.	Questionnaire	Good intake of oil (86%), meat/fish (92%), and fruits/vegetables (56%), and low prevalence of pica (17%). No significant taboos.	All participants believed in eating more during pregnancy for healthy neonates; no taboos like avoiding food to prevent large neonates were reported.	Despite limited education and a rural setting, participants demonstrated healthy nutritional practices and did not adhere to harmful food taboos.
Walters et al. (2019) [40]	Quantitative	Malawi – Msundwe (rural)	62	The mean age of the participants was 17.7 years; most were married adolescents with primary education and were primigravida.	Questionnaire	Low dietary diversity; no dairy consumed; 7% consumed eggs. Less than half of the participants (39%) reported consuming meat, poultry, or fish. The main foods consumed were nsima, vegetables, and fruits.	35% reported food taboos. Eggs → bald neonates; pork/okra/groundnuts/cabbage /sugarcane → taboos	Nutrition in rural pregnant adolescents was poor. Calls for antenatal education on diet and taboos.
Oko-Ose et al. (2021) [41]	Quantitative	Nigeria – Edo State (urban)	284	The majority of the participants were aged 25-35 years old and were married (90%). A total of 46.5% had a tertiary education level.	Questionnaire	Good general nutritional intake; low intake of protein- and calcium-rich foods	Cultural taboos influence food avoidance. Not specified motivations behind dietary practices.	Although knowledge and awareness of healthy nutrition were high, cultural beliefs continued to influence pregnant women’s dietary practices. Health education should place greater emphasis on promoting healthy nutrition.
Afolabi et al. (2024) [42]	Quantitative	Nigeria – Osun State (urban)	189	The majority of participants were aged 26-30 years and married (60%). A total of 34.9% had completed secondary education, and 52.9% had experienced three previous pregnancies.	Questionnaire	Moderate food intake overall; low protein/micronutrient intake. Dietary avoidance of snails, okra, and cocoa drinks.	Beliefs that eating less eases delivery: snails → drooling; okra → dullness; cocoa → unsafe.	Food taboos and traditional beliefs relating to pregnancy exist, and a significant proportion of women still believe in old, unscientific tales. Nutrition counseling is key.
Akiso et al. (2024) [43]	Quantitative	Ethiopia – Durame Town (rural and urban)	422	The participants had a mean age of 30.5 years; the majority were married (98.8%); 45.5% were housekeepers; 30.6% had three to six children; and 36% were college educated.	Questionnaire	About half (54.5%) of the participants represented avoidance of nutrient-rich foods: eggs, fatty meat, milk, honey, vegetables, and fruits.	Fears of difficult labor, miscarriage, and fetal complications.	Several factors (i.e., age, family size, previous antenatal care follow-up, etc.) were found to have an impact on food taboo practice.
Oni and Tukur (2012) [44]	Mixed methods	Nigeria – Oyo State (rural)	405	The majority (31.1%) were aged 20-24 years, had a primary educational level (65.7%), and had 1-2 children (34.1%). A total of 13.3% had food taboos in pregnancy.	FGDs and questionnaire	Dietary avoidance of snails, okra, bush animals, and cocoa beverages	Snails → drooling; bush animals → evil spirits; cocoa → large newborns and complications during childbirth	Health professionals should assess food taboos during pregnancy.
Tugume et al. (2024) [45]	Mixed methods	Uganda – Buyende District (rural)	256	The majority of the participants were aged 21-30 years, were married (88.1%), had a primary education (74.9%), and were farmers.	FGDs and questionnaire	Dietary avoidance of sugarcane, fish, pineapple, oranges, eggs, and chicken	Beliefs linked to abortion, fetal macrosomia, birth complications, skin issues, and drooling.	Food taboos among women were deeply rooted in cultural beliefs and posed a barrier to adequate maternal nutrition.

Results of Individual Sources of Evidence and Synthesis of Results

Regarding the described dietary practices, only one study [39] reported observing no significant food taboos. Authors pointed out that despite limited education and a rural setting, participants demonstrated healthy nutritional behaviors and did not adhere to harmful cultural dietary restrictions [39].

Avoidance of protein-rich foods: The majority of studies (11 out of 13) [16,35-38,40-45] reported that participants avoided protein-rich foods. All but one study [41] specified particular foods that were avoided. Meat was the most frequently reported avoided food, mentioned in eight studies: bush animals [44], grasscutter meat [16], general references to meat [34], solid/fatty meat [35,43], pork [37,40], duck meat [37], and chicken [37,45]. Eggs were the second most commonly avoided food, as they were reported in six studies [36-38,40,43,45]. The third most frequently avoided food was snails, reported in three studies [16,42,44].

Avoidance of dairy, vegetables, and fruits: Five out of the 13 studies reported that participants avoided dairy products [34-36,40,43], while one study [41] reported only a low intake of calcium-rich foods. Avoidance of okra was noted in three studies [40,42,44]. Other vegetables mentioned in more than one study included green pepper [34,38] and cabbage [36,40]. Fruit avoidance, either specifically or in general, was reported in four studies [36,37,43,45].

Avoidance of other food taboos: Among other reported food taboos, sugarcane was the most frequently avoided, mentioned in four studies [34,36,40,45]. Cocoa was reported as avoided in two studies [42,44]. The avoidance of groundnuts was noted in one study [40], while mustard [38] and honey [43] were each reported in one study, respectively.

Cultural Motivations

The most common reason for avoiding all of the abovementioned foods was the belief that they could lead to complications during labor or negatively affect the newborn. Some studies did not provide detailed explanations of the specific beliefs associated with the avoidance of each food item. The labor-related complications mentioned in a general context included bleeding during labor, childbirth complications, delayed childbirth, and difficult labor, as reported in several studies [35,36,38,43,45]. The following foods were specifically linked to labor-related complications: cocoa, believed to cause complications during childbirth [44]; grass-cutter meat, thought to lead to prolonged labor [16]; and sugarcane and fruits, associated with increased labor risks [34]. The neonatal complications reported in a general context included miscarriage, fetal complications, plastered fetal heads, fetal macrosomia, large neonates, skin discoloration, and other skin issues [35,36,38,43,46]. The following foods were associated with specific beliefs regarding neonatal complications: snails, believed to cause drooling in newborns [16,42,44]; eggs, thought to result in bald neonates [37,40]; cocoa, believed to cause large newborns and considered unsafe [44,42]; leafy greens and dairy products, thought to cause food to stick to the fetus’ head [34]; pork, believed to result in newborns crying like pigs [37]; and red fruits and vegetables, which were believed to cause the newborn to be born with a red anus [37].

Discussion

The objective of this scoping review was to explore African women’s cultural and traditional food practices during pregnancy and explore the motivations behind these practices. Thirteen studies, out of five African nations, such as Nigeria, Ethiopia, Malawi, Democratic Republic of the Congo, and Uganda, published during the years 2012-2024 were included. Results affirm that the sociocultural beliefs surrounding food taboos continue to shape the nutritional practices adopted by pregnant African women. Food taboos are not exclusive to African nations; they are also found in culturally diverse countries such as those in some parts of Asia [17,46-51].

According to the mapping of dietary taboos, the most common tendency among the research was avoiding foods rich in protein, such as meat, eggs, chicken, fish, dairy products, and snails [16,34-38,40-45]. Similar findings have also been documented in other cultural settings, including China and India [48,49]. The most frequently reported reason for avoiding the aforementioned protein-rich foods was the belief that they could cause complications during labor or have negative effects on the newborn. Particularly interesting is the case of snails, which are believed to cause excessive drooling in newborns [16,42,44]. However, this belief is refuted by scientific data. On the contrary, the giant African snail (*Archachatina marginata*) has been shown to be a rich source of protein, trace elements, and minerals essential for healthy growth and development in humans [52]. Maintaining a successful pregnancy requires more than just consuming foods that contain protein; it requires additional protein intake. Extra dietary protein and amino acids are essential to support the physiological changes that occur during pregnancy, such as the expansion of maternal tissues and blood volume, as well as the growth and development of the fetus and placenta. Significant protein demands are placed on the pregnant woman by the growth of new fetal and placental tissues as well as the rise in maternal body mass [53]. Thus, some of the adverse impacts of inadequate maternal protein intake include decreased histotroph secretion, delayed embryonic development, and diminished placental angiogenesis, growth, and function. It can also cause a variety of nutrient deficiencies, disrupt the transport and absorption of fat-soluble vitamins, lipids, and essential microminerals like iron and zinc, and hinder the supply of nutrients from the mother to the infant. IUGR, pregnancy problems, fetal loss, and poor maternal health are all possible outcomes of these impacts [54,55].

Pregnant women in Africa avoided vegetables and fruits as the second main category of foods [34,36-38,40,42-45]. This finding has also been observed in cultural contexts across Asia [49,51,56]. The most common reason given for not consuming vegetables and fruits was the same as that for protein foods, i.e., beliefs that they could lead to complicated labor or adversely affect the newborn. Nutrient-dense fruits and vegetables are important sources of many components. They are high in vitamins like folate, C, and A; dietary fiber; antioxidants; and key minerals like potassium and magnesium. To maintain the physiological changes of pregnancy and lessen labor problems, these nutrients must be consumed. Micronutrients found in fruits and vegetables have been shown to promote good placental and immune system function, both of which are essential for a fetus’s healthy growth [57-59]. Several studies have connected improved childbirth and neonatal outcomes to increased fruit and vegetable intake during pregnancy. Specifically, diets high in fruits and vegetables have been linked to a reduced risk of preterm birth and small-for-gestational-age infants [60,61].

In summary, the mapping from the present scoping review revealed that the main categories of food taboos that are avoided by pregnant African women are those that are rich in protein, as well as vegetables and fruits. This finding contradicts the recommendations of WHO, which state that pregnancy requires a healthy diet that includes an adequate intake of energy, protein, vitamins, and minerals obtained through the consumption of a variety of foods, such as green and orange vegetables, meat, fish, beans, nuts, whole grains, and fruit, to meet both maternal and fetal needs [6]. According to the Academy of Nutrition and Dietetics, eating a wide range of meals and taking the right vitamins and minerals are two factors that contribute to a healthy pregnancy result. These findings also contradict this recommendation. It is also underlined that insufficient amounts of essential nutrients during crucial stages of fetal development could cause reprogramming in the tissues of the fetus, which could put the child at risk for chronic illnesses in the future [62]. Accordingly, the National Health Service and the American College of Obstetricians and Gynecologists recommend balanced maternal meals full of fruits, vegetables, proteins, and vital micronutrients, along with certain supplements [63,64].

The second finding of the present study, which was also oriented toward the second research question, is that mapping specifically centered on why certain foods were avoided by pregnant African women. The reasons lay in cultural motivations, which were primarily linked to fears about having a negative impact on pregnancy, such as miscarriage, difficult labor, or health problems concerning the newborn. It should come as no surprise that cultural beliefs have a significant impact on pregnant women’s food choices and dietary practices since culture encompasses the values, beliefs, lifestyles, and traditions that are passed down from generation to generation. Consequently, dietary taboos and limits may be maintained as deeply ingrained social norms that influence maternal behavior in ways that are culturally significant rather than just being personal preferences. However, the issue arises when cultural norms prevent pregnant women from receiving adequate nutrition [65-67]. In order to achieve adequate nutrition during pregnancy, it is essential to appropriately address food taboos. Implementing targeted actions, such as nutrition education and counselling, constitutes an intervention recommended by reputable scientific organizations [68,69]. Moreover, nearly all the authors of the studies included in the present review explicitly recommend nutrition education and counselling during pregnancy for pregnant African women [16,34-42,45]. Maternal nutrition counselling supports women and their families in making informed decisions and taking action to improve nutritional health. This includes recommendations for the types, categories, and amounts of food that a pregnant woman should eat in order to fulfill her nutritional demands, as well as for the right amounts of physical exercise and the appropriate use of dietary supplements [68,69]. For nutrition education and counselling to achieve the desired outcomes, healthcare professionals need to apply several principles, such as cultural awareness and cultural knowledge. Cultural awareness involves learning about and understanding a person’s culture as well as how it affects their values, beliefs, behavior, and problem-solving approaches. Cultural knowledge means becoming familiar with cultural differences in family structure, health beliefs, and general sociodemographic characteristics of culturally diverse groups [70]. This strategy will give pregnant African women a sense of understanding and respect for their values, beliefs, and behaviors.

The current study significantly advances the field of research on the nutrition of pregnant women who avoid certain foods. This study’s focus on pregnant African women offers a chance to increase our understanding of this particular population, whose migration to Europe cannot be ignored. The current scoping review added to the body of knowledge on maternal health worldwide by identifying cultural and/or traditional dietary patterns among pregnant African women and investigating the belief-based justifications for these practices. Furthermore, the results of this study could serve as a source of mapping information regarding nutrition for midwifery experts who interact with pregnant African women in a clinical context.

This study has potential limitations. Since the search was conducted across three databases, more information that would have contributed to the most valid conclusion was overlooked. The fact that the included studies were on pregnant African women within the African continent is an additional limitation. Therefore, information for pregnant African women living outside of Africa cannot be reliably provided by the current review. This is an important issue for future research. Whether pregnant African women truly uphold the food taboos they have when living outside of Africa requires more research. If they don’t, it would be intriguing to find out if nutritional counseling or their assimilation into their new cultural environment is responsible for the change in attitude. Furthermore, it will greatly advance our understanding in this field if future studies investigate the connection between pregnant African women’s dietary taboos and their health beliefs.

## Conclusions

Food taboos among African pregnant women include two main food categories. Foods that are high in protein come first, followed by fruits and vegetables. These food limitations are upheld for the following reasons: worries and fears about potential adverse effects on pregnancy or childbirth, as well as worries about potential health issues for the newborn. African pregnant women’s food taboos are a result of a complex interaction between traditional knowledge, cultural beliefs, and communal norms. Healthcare professionals in midwifery clinical settings need to approach these dietary practices with cultural sensitivity. Empowering pregnant African women with accurate knowledge and culturally relevant advice can help them maintain a balance between tradition and the needs for maternal and child well-being.
